# High mannose-binding *Pseudomonas fluorescens* lectin (PFL) downregulates cell surface integrin/EGFR and induces autophagy in gastric cancer cells

**DOI:** 10.1186/s12885-016-2099-2

**Published:** 2016-02-06

**Authors:** Yuichiro Sato, Takanori Kubo, Kinjiro Morimoto, Kazuyoshi Yanagihara, Toshio Seyama

**Affiliations:** Department of Medical Pharmacy, Faculty of Pharmacy, Yasuda Women’s University, Hiroshima, Japan; Department of Life Sciences, Faculty of Pharmacy, Yasuda Women’s University, Hiroshima, Japan; Present address; Division of Translational Research, National Cancer Center Research Institute, 6-5-1 Kashiwanoha, Kashiwa, Chiba Japan

**Keywords:** *Pseudomonas fluorescens* lectin, Integrin, EGFR, Autophagy

## Abstract

**Background:**

*Pseudomonas fluorescens* lectin (PFL) belongs to a recently discovered anti-HIV lectin family and induces anoikis-like cell death of MKN28 gastric cancer cells by causing α2 integrin internalization through recognition of high mannose glycans; however, the detailed anti-cancer mechanism is not fully elucidated.

**Methods:**

Cell adherence potency of MKN28 upon PFL treatment was assessed using a colorimetric assay. Cell surface molecules to which PFL bound were identified by peptide mass finger printing with Matrix Assisted Laser Desorption/Ionization-time of flight mass spectrometry and their cellular localization determined by immunofluorescence microscopy. Gene and protein expression in PFL-treated MKN28 cells were evaluated by microarray analysis and western blot, and the function of these genes was evaluated by siRNA knock-down. A proliferation assay measured the sensitivity of PFL-treated cancer cells to anti-cancer drugs. The effect of PFL on subcutaneous MKN28 tumor growth and hepatic tumor formation in BALB/c nude mice was evaluated.

**Results:**

The strength of MKN28 cell adherence in vitro to the extracellular matrix was impaired by PFL treatment, consistent with the observation that PFL induces rapid downregulation of surface integrins. PFL also was found to bind to cell surface epidermal growth factor receptor (EGFR). Surface EGFR molecules were endocytosed following PFL binding, and were degraded in a time-dependent fashion. This degradation process was largely the result of autophagy, as revealed by the increased expression of autophagic proteins. PFL-induced EGFR degradation was partly inhibited by *RAB7* siRNA as well as *LC3* siRNA, and internalized EGFR colocalized with ATG9 at 48 h post-PFL treatment, suggesting that these proteins contribute to dynamic degradation induced by PFL. PFL-induced decrease in surface EGFR rendered MKN28 cells susceptible to gefitinib, a selective inhibitor of EGFR tyrosine kinase. In vivo experiments showed that PFL-treated MKN28-EGFP cells injected in the portal vein of BALB/c nude mice failed to form tumor colonies on the liver, and intratumoral injection of PFL significantly inhibited tumor growth.

**Conclusion:**

PFL-mediated downregulation of integrin and EGFR contributes to the inhibition of tumor growth in vitro and in vivo. This novel anti-cancer mechanism of PFL suggests that this lectin would be useful as an anti-cancer drug or an adjuvant for other drugs.

**Electronic supplementary material:**

The online version of this article (doi:10.1186/s12885-016-2099-2) contains supplementary material, which is available to authorized users.

## Background

Lectins, carbohydrate-binding proteins, are distributed ubiquitously in a variety of organisms. Among them, high mannose-binding lectins have attracted attention for biomedical research as promising biomolecules with anti-viral or anti-tumor activities [1]. Recently, a novel high mannose-binding lectin family has been discovered in lower organisms such as bacteria, cyanobacteria and marine algae [2]. This family includes well-characterized cyanobacterial OAA from *Oscillatoria agardhii* [2, 3], red algal ESA-2 from *Eucheuma serra* [4] and KAA-2 from *Kappaphycus alvarezii* [5], bacterial PFL from *Pseudomonas fluorescens* Pf0-1 [6], and several other homologous proteins [7]. These proteins commonly show exclusive specificity for high mannose N-glycans but no monosaccharide-binding [2, 4–6]. At low nanomolar levels, some of these lectins exhibit potent anti-viral activity against HIV and influenza viruses, through recognition of high mannose glycans on virus envelope glycoproteins [2–6]. At micromolar or higher concentrations, some lectins, such as ESA-2 and PFL, show cytotoxicity for various cancer cells [6, 8]. It has been proposed that the cell death of colon carcinoma Colon26 cells induced by ESA-2 is mediated by the apoptosis pathway [8]. By contrast, our recent study demonstrated that PFL induces anoikis-like cell death of MKN28 human gastric cancer cells via interaction with cell surface integrin molecules [6]. This cell death that accompanies loss of cell adhesion was presumably due to the rapid internalization of cell surface integrins upon direct binding of PFL to high mannose glycans on α2 integrin; however, the detailed mechanism of death signaling has not been fully elucidated.

In this study, we have further explored the PFL target molecule(s) on MKN28 cells and identified the involvement of epidermal growth factor receptor (EGFR). Intriguingly, similar to the dynamic redistribution of integrins, cell surface EGFR also internalized to the cytoplasm following PFL treatment. We therefore investigated the adjuvant effect of PFL in combination with EGFR inhibiting anti-cancer drugs currently in clinical use. Moreover, to address the anti-cancer mechanism induced by PFL in more detail, the participation of the autophagy and apoptosis pathways was analyzed. Finally, the *in vivo* effect of PFL was evaluated using a subcutaneous MKN28 tumor model.

## Methods

### Materials

The antibodies against autophagy-related proteins (Beclin1, ATG3, ATG 5, ATG 7, ATG 9A, ATG 12, ATG 13, LC3, and HSPB8), apoptosis-related proteins (caspase-9, cleaved caspase-9, caspase-7, cleaved caspase-7, caspase-3, and cleaved caspase-3) and RAB7 were purchased from Cell Signaling Technology (CST, Japan). The anti-actin antibody was purchased from Santa Cruz. The Alexa-conjugated anti-mouse and anti-rabbit IgG were purchased from Life Technologies. Small interfering RNA (siRNA) for *ATG9A*, *HSPB8*, *LC3*, *RAB7* and non-targeting control siRNA were purchased from Thermo Scientific (Lafayette, CO). siRNAs for *Beclin1* and *ATG5* were purchased from CST. Gefitinib was purchased from Cayman chemical (MI).

### Cell lines and culture conditions

The gastric cancer cell line MKN28 was maintained in RPMI-1640 medium (Wako, Japan) supplemented with 10 % fetal bovine serum (FBS, GIBCO), 100 IU/mL penicillin G sodium, and 100 μg/mL streptomycin sulfate as described previously [6]. Both MKN28-EGFR and the human colon cancer cell line HT-29 Luc cells were obtained from Dr. Yanagihara at National Cancer Center Research Institute, and were maintained as described above.

### In vitro cell adhesion assay

The effect of PFL on cell adhesion was evaluated using the CytoSelect cell adhesion assay (Cell Biolabs, San Diego, USA). MKN28 cells were treated with 10 μM PFL for 3 h in RPMI-1640 medium with 10 % FBS. The cell suspensions were allowed to attach to an ECM-coated 48-well plate for 90 min at 37 °C. Adherent cells were stained and extracted according to manufacturer’s protocols. Extracted samples were measured at 560 nm in a plate reader (1420 multilabel counter, PerkinElmer).

### Isolation of PFL-binding molecule(s) from MKN28 cell

The cell surface molecule(s) that interacted with PFL were identified as described previously [6]. Briefly, biotinylated-PFL (200 μg) was added to confluent MKN28 cells and incubated for 2 h at room temperature. The cells were lysed in 800 μL of RIPA buffer (50 mM Tris–HCl, pH 7.6, 150 mM NaCl, 1 % Nonidet P40, 0.5 % sodium deoxycholate, protease inhibitor cocktail, 0.1 % SDS). Subsequently, 100 μL of biotin-capture avidin beads (Adar Biotech, Israel) was added to the cell lysate and incubated overnight at 4 °C with gentle agitation. Captured proteins on beads were eluted with 50 μL of SDS-PAGE sample buffer (62.5 mM Tris, pH 6.8, 2 % SDS, 10 % glycerol, 1 % mercaptoethanol, and 0.003 % bromphenol blue) for 15 min at 90 °C and subjected to SDS-PAGE. The protein band specific for the PFL treatment fraction was analyzed by Matrix Assisted Laser Desorption/Ionization-time of flight mass spectrometry (MALDI-TOF MS) after in-gel digestion with trypsin. The peptide mass finger printing data was searched using Mascot software (Matrix Science, Japan).

### Cellular localization of α2 integrin and EGFR

The cellular distribution of EGFR upon PFL treatment was observed by immunofluorescence microscopy as described previously [6]. Confluent MKN28 cells growing on coverslips in a 6-well plate were treated with 30 μg/mL Alexa488-conjugated-PFL in RPMI-1640 and incubated for various periods of time. The cells were fixed with 80 % acetone and incubated with mouse monoclonal anti-EGFR antibody (Thermo Scientific, UK) at 37 °C for 1 h. Subsequently, the cells were incubated with Alexa568-conjugated goat anti-mouse IgG antibody (Life technologies, Japan) at 37 °C for 1 h. The cells were mounted using Vectashield with DAPI (Vector Laboratories) and were observed using a confocal laser scanning microscope (IX70; Olympus, Japan). The cellular distribution of EGFR together with ATG9 was examined in a similar way using mouse anti-EGFR antibody and rabbit anti-ATG9 antibody. Accordingly, a distinct secondary antibody was employed to visualize EGFR and ATG9, using Alexa488-conjugated goat anti-mouse IgG antibody and Alexa568-conjugated goat anti-rabbit IgG antibody, respectively. The cellular distribution of α2 integrin following PFL treatment was observed as above after incubation with 10 μM PFL for 24 h.

### Western blotting

Expression of cellular proteins related to the autophagy and apoptosis pathways were determined by western blotting. Confluent MKN28 cells cultured in 6-well plates were incubated with 2 μM PFL in RPMI-1640 with FBS for 3, 24, 48 and 72 h, and the cells were lysed with 800 μL of RIPA buffer for 30 min on ice with gentle shaking until the cells were completely lysed. Portions of each sample were applied to SDS-PAGE and visualized by western blotting. The proteins were detected using a specific rabbit antibody (1:1000) against each protein. A horseradish peroxidase (HRP)-conjugated anti-rabbit IgG (1:10,000) was used to visualize the signal by ECL prime (GE healthcare, UK).

### Adjuvant effect of PFL for anti-cancer drugs

The susceptibility of MKN28 cells to gefitinib was evaluated by measuring cell proliferation using the CellTiter 96 cell proliferation assay (Promega, Madison, WI). Cells seeded on 96-well microplates were pretreated with 2 μM PFL for 24 h. The cells were washed once with RPMI-1640 and incubated for 72 h with various concentrations of gefitinib in RPMI-1640 medium with 10 % FBS. The cells were subsequently incubated with 20 μL of MTS reagent for 1 h at 37 °C and measured with a microplate reader (1420 multilabel counter, PerkinElmer) at 490 nm. The sensitivity of another colon cancer line, HT-29 Luc, to 5-Fluorouracil (5-FU) also was tested to evaluate the effect of co-incubation of anti-cancer drugs with PFL. These cells were incubated with various concentrations of 5-FU for 96 h in the presence or absence of 0.5 μM PFL, and the cell survival ratio was determined by CellTiter-Glo luminescent cell viability assay (Promega, Madison, WI).

### DNA microarray analysis

MKN28 cells were incubated with 2 μM PFL in RPMI-1640 containing 10 % FBS for 24, 48, and 72 h, and PFL-untreated cells were incubated for 24 h without PFL (as a control for comparing gene expression level). Total RNA from the cells was extracted with the RNeasy kit (QIAGEN, Valecia, CA) and 100 ng of each RNA was subjected to Aligent Expression Array Analysis with SurePrint G3 Human GE 8x60K v2 (Takara Bio, Japan). The expression levels of genes at each time were determined and compared with those of the control RNA from PFL-untreated cells.

### Effect of siRNAs on PFL-induced EGFR degradation

MKN28 cells cultured in 24-well plates were transfected with 100 nM siRNA in non-serum RPMI-1640 using DharmaFECT transfection reagents (Thermo Scientific, Lafayette, CO). At 24 h post-transfection, the culture medium containing the siRNA was removed and the cells were washed once with RPMI-1640. Then, the cells were incubated with 2 μM PFL in RPMI-1640 with FBS for 72 h, and cell extracts for western blotting were prepared as described above. Expression of EGFR was assessed by incubating with rabbit anti-EGFR antibody (CST, Japan) followed by HRP-conjugated anti-rabbit IgG (CST, Japan).

### Mice

Animal experiments were approved by the animal ethics committee of the Yasuda Women’s University (approval number: 1120) and carried out under guidelines to minimize suffering. Experimental mice were housed in an approved clean facility, in a room with a controlled light/day cycle and temperature.

### In vivo tumor formation assay

Seven-week-old female BALB/c nude mice (Clea Japan) were bred under SPF conditions. MKN28-EGFP cells (1 × 10^7^ cells/mL) in 100 μL RPMI-1640 were injected into the portal vein to implant the tumor cells. In PFL-treated groups, the cells were pre-incubated with 10 μM PFL for 3 h in RPMI-1640 with 10 % FBS followed by washing with the same medium without the lectin. The tumor colonies, formed in the liver 4 weeks after tumor implantation, were observed directly or using an *in vivo* imaging system (IVIS; Xenogen, LUMINA II, CA) after dissection. *In vivo* tumor formation experiments were performed according to the guidelines for animal experiments established by Yasuda Women’s University.

### In vivo effect of PFL on tumor growth in nude mice

The experiment was performed according to the guidelines for animal experiments established by Yasuda Women’s University. Six-week-old female BALB/c nude mice (Clea Japan) were bred under SPF conditions. MKN28-EGFP cells (1 × 10^7^ cells) in 100 μL RPMI-1640 were subcutaneously injected at both ventral regions of the mice to implant the tumor cells. Intratumoral injection of PFL (1 mg/mL) in 100 μL phosphate buffered saline (PBS) was performed 1, 3, and 7 days after tumor cell implantation. The same volume of PBS was used as a vehicle control. The length and width of the implanted tumor mass were measured every third day following the initial injection, and the tumor volume was estimated.

## Results

### PFL changes cell adherence strength by inducing intracellular trafficking of integrin

**Fig. 1:**
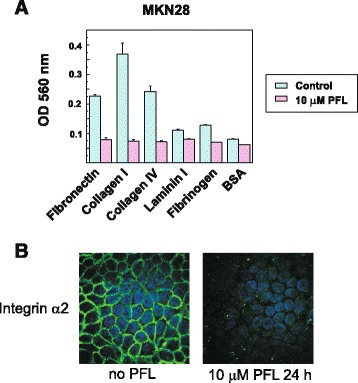
**a** Change of adhesive properties of cells to ECM after PFL treatment. MKN28 cells were treated with 10 μM PFL for 3 h and spread on ECM-coated plates. The cells were allowed to attach to ECM-coated plates for 90 min at 37 °C. Attached cells were detected by a colorimetric method. Controls were evaluated in the same way without PFL. **b** Down regulation of surface α2 integrin in MKN28 cells after a 24 h incubation with 10 μM PFL. Cellular distribution of α2 integrin after PFL treatment was observed by immunofluorescence microscopy using anti- α2 integrin antibody

Our previous study demonstrated that the bacterial lectin PFL induces intracellular trafficking of α2 integrin in MKN28 gastric cancer cells through the specific recognition of high mannose glycans on α2 integrin [6]. We therefore tested the extracellular matrix (ECM)-binding profiles of MKN28 cells after PFL treatment using a colorimetric assay and ECM-coated plates. In the absence of PFL, MKN28 cells attached strongly to collagen type I and IV, and to fibronectin (Fig. 1a). However, after 10 μM PFL treatment for 3 h, the adhesive properties of MKN28 cells to these ECMs were completely abolished, as shown in Fig. 1a. The α2 integrin molecules on the cell surface were significantly down-regulated by treatment with 10 μM PFL for 24 h (Fig. 1b). The loss of cell adherence strength caused by PFL is well correlated with the rapid down regulation of cell surface integrins, which has been previously described [6].

### Identification of cell surface molecules targeted by PFL

**Fig. 2:**
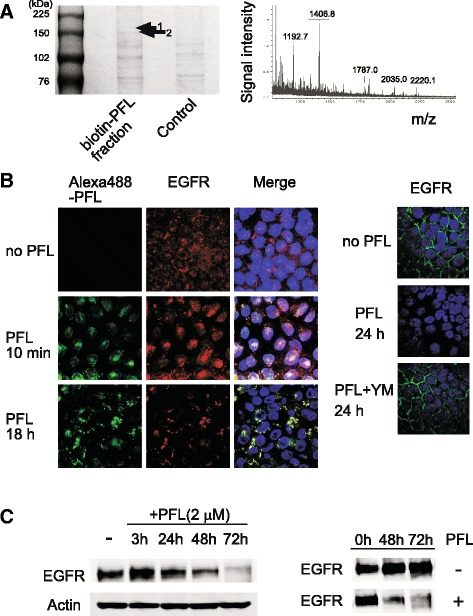
**a** Identification of a cell surface molecule that interacts with PFL on MKN28 cells. The cells were treated with biotin-PFL and proteins that bound to biotin-PFL were precipitated with avidin-coated beads. Proteins captured on beads were separated by SDS-PAGE (left panel) and the protein specific for the PFL fraction (arrow 1) was analyzed by MALDI-TOF MS after in-gel digestion with trypsin. The peptide mass finger printing data (right panel) thus obtained was searched using Mascot software. **b** Effect of PFL on cellular distribution of EGFR. The change of cellular localization of EGFR induced by PFL was observed using confocal fluorescence microscopy. The distribution of Alexa-488 PFL (green) and EGFR (red) are shown. Colocalization of Alexa-488 PFL and EGFR is shown as a yellow signal (Merge). Nuclei within the cells were stained with DAPI. In the right panel, the effect of yeast mannan (YM) on PFL-induced trafficking of EGFR is shown. The change in distribution of EGFR (green) was observed by confocal fluorescence microscopy. **c** Western blots show the protein levels of EGFR in MKN28 cells upon treatment with 2 μM PFL for 3, 24, 48, and 72 h and without treatment (shown as -). In the right panel, western blots show the protein levels of EGFR in MKN28 cells determined in the presence or absence of 2 μM PFL

In our previous study, we demonstrated that biotinylated PFL interacts directly with high mannose glycans on α2 integrin expressed on the surface of MKN28 cells [6]. To further explore the cell surface molecule(s) to which PFL bound, biotin-PFL was incubated with MKN28 cells and the molecules bound to biotin-PFL were co-precipitated with avidin-coated beads. The proteins trapped on the beads were analyzed by SDS-PAGE and the specific band in the PFL fraction (Fig. 2a, left panel, arrow 1 or 2) was identified by peptide mass fingerprinting using MALDI-TOF mass spectrometry after in gel digestion with trypsin. We searched a database for the peptide mass data obtained from the band identified by arrow 1 (Fig. 2a, right panel). The band, with a weight of 170 kDa (arrow 1) was identified as EGFR, with a probability score of 92 (*p* < 0.05). The 150 kDa band (arrow 2) was α2 integrin, as was previously identified [6].

### PFL induces intracellular trafficking of EGFR

Cellular localization of EGFR upon PFL treatment was then examined using confocal fluorescence microscopy. Fluorescent-labeled PFL (Alexa488-PFL) was used to track PFL localization. EGFR was detected with an anti-EGFR monoclonal antibody followed by Alexa568-conjugated secondary antibody. As shown in Fig. 2b (left panel), PFL induced rapid trafficking of surface EGFR to the cytoplasm. Treatment with PFL for 10 min induced internalization of EGFR, and the EGFR seemed to colocalize with PFL. PFL-induced EGFR trafficking was inhibited effectively by yeast mannan, a glycoprotein bearing high mannose glycans, suggesting the interaction between PFL and EGFR was mediated by these glycans (Fig. 2b, right panel). Western blot revealed that the bands corresponding to EGFR were diminished in a time-dependent fashion in the presence of PFL (Fig. 2c), suggesting the contribution of some degradation process, such as autophagy after PFL-induced EGFR internalization. The protein level of EGFR was only reduced in the presence of PFL, as shown in Fig. 2c (right panel).

### PFL induces autophagy but not apoptosis in MKN28 cells

**Fig. 3:**
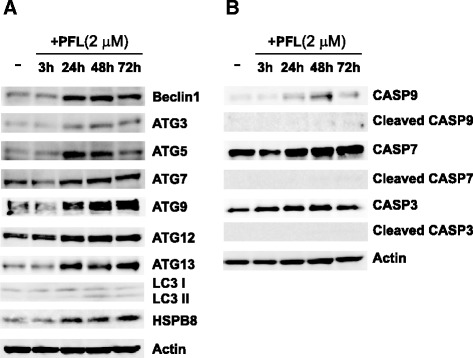
Change of expression levels of autophagy or apoptosis related proteins in MKN28 cells after PFL treatment. After treatment with 2 μM PFL for the indicated time, the cells were lysed and subjected to immunoblot analysis. Western blots show the protein expression detected for autophagy-related proteins (**a**) and apoptosis-related proteins (**b**)

To determine whether PFL stimulates autophagy in MKN28 cells, expression levels of autophagy-related proteins were estimated by western blotting. MKN28 cells were incubated with 2 μM PFL in RPMI-1640 medium with 10 % FBS for 3–72 h. As shown in Fig. 3a, time dependent increase of Beclin-1, Autophagy Related 3(ATG3), ATG5, ATG7, ATG9A, ATG12, and ATG13 was observed. The expression of ATG13, its phosphorylation state contributes to autophagy induction, has been increased at early stage, 24 h after PFL treatment. Similarly, Beclin-1 and ATG5, which are important for vesicle nucleation and vesicle elongation, respectively, showed increased expression 24 h after PFL treatment. The autophagic marker LC3II, which is converted from LC3I by the sequential action of ATG7 and ATG3, also appeared at a relatively late stage, 48 h after PFL treatment. The expression of HSPB8, a member of the small heat shock protein (HSP) family that stimulates protein degradation by macroautophagy, was also increased by PFL treatment. Consistent with increased expression of autophagy-related proteins, the expression of autophagic genes also was enhanced by PFL, as confirmed by microarray analysis (Table 1, original data is available in Additional file 1: Table e1). These data suggested that the internalized PFL-EGFR complex triggered macroautophagy or chaperone-mediated autophagy after being delivered to a specific cell compartment. Conversely, PFL did not activate the apoptosis pathway, as shown in Fig. 3b, since no cleaved forms of caspase-9, caspase-7 nor caspase-3 were detected.

**Table 1:**
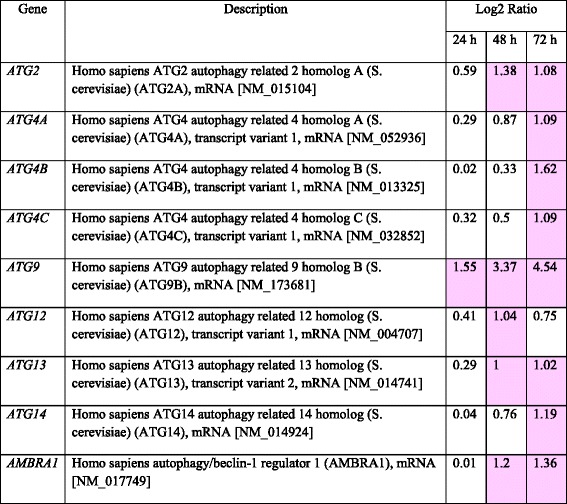
Change of expression of autophagy related genes in MKN28 cells after PFL treatment

Total RNA from PFL-treated and untreated MKN28 cells was extracted and subjected to Aligent Expression Array Analysis with SurePrint G3 Human GE 8x60K v2 (Takara Bio, Japan). The expression level of each gene at each time was determined and compared with that of control RNA from PFL-untreated cells. Highlighting indicates more than a 2-fold change in the expression level.

**Fig. 4:**
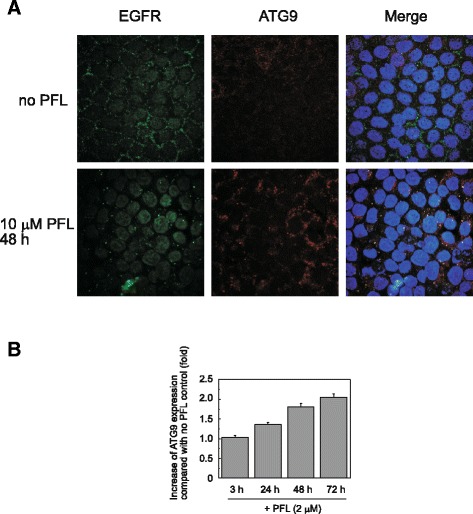
Involvement of ATG9 in PFL-induced intracellular trafficking of EGFR. **a** MKN28 cells were incubated with 10 μM PFL for 48 h. EGFR and ATG9 were visualized by confocal fluorescence microscopy. Representative photomicrographs show the cellular localization of EGFR (green) and ATG9 (red); colocalization of both proteins is represented as a yellow signal (merged panel). **b** Histograms represent the expression of ATG9 in MKN28 cells after 2 μM PFL treatment. Protein expression was determined by immunoblot analysis

A substantial amount of internalized EGFR was observed as assembled small particles 48 h after PFL treatment, as shown in Fig. 4a. ATG9A, an integral membrane protein required for autophagosome formation, was induced by PFL treatment and colocalized with EGFR. The protein expression level of ATG9A at 48 h was increased, as judged from the intensity of red fluorescence compared to that of the PFL-untreated control cells, consistent with the results of western blotting (Figs. 3a and 4b).

#### RAB7 is essential for PFL-induced EGFR degradation

**Fig. 5:**
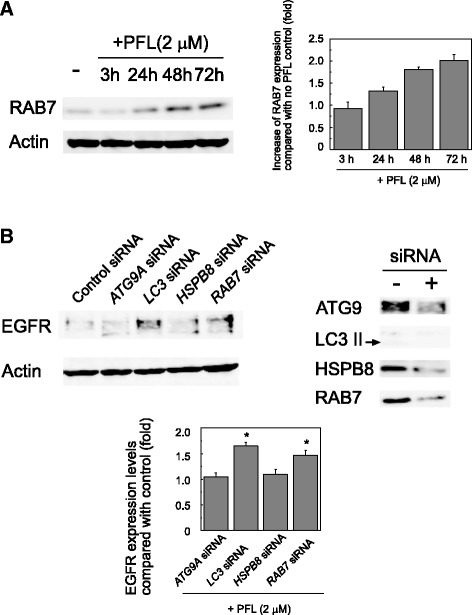
Involvement of RAB7 in PFL-induced EGFR degradation. **a** Western blots show the protein expression of RAB7 in MKN28 cells treated with PFL. The cells were incubated with 2 μM PFL in RPMI-1640 with FBS for the indicated time and protein expression was analyzed and quantified by immunoblotting. **b** Inhibition of EGFR degradation induced by PFL with *RAB7* siRNA. MKN28 cells were incubated with various siRNAs against *ATG9A, LC3, HSPB8,* and *RAB7* in RPMI-1640 without FBS for 24 h and subsequently incubated with 2 μM PFL in RPMI-1640 with FBS. As a reference, non-targeting control siRNA was used. Western blots show the EGFR expression for each sample (left panel) and the knock-down efficiency of each siRNA (right panel). The histogram (bottom panel) displays the quantified expression of EGFR from each condition; asterisks indicate values that were significantly different (*p* < 0.05) from control as revealed by one way ANOVA

RNA expression of the RAB family proteins, considered molecular switches of membrane traffic, were found to be up- or down-regulated in PFL-treated MKN28 cells by microarray analysis (Table 2). Among them, expression of *RAB7*, which is known as a common modulator in endocytosis and autophagy, was increased by the presence of PFL (Table 2). Consistent with this observation, the protein level of RAB7 was elevated significantly in PFL-treated MKN28 cells (Fig. 5a). PFL treatment triggered *de novo* synthesis of a key factor of membrane traffic and autophagy. We therefore tested the effect of *RAB7* siRNA on PFL-induced EGFR degradation. As shown in Fig. 5b, PFL-induced degradation of EGFR was partially rescued by *RAB7* siRNAs, as well as by *LC3* siRNA, but not *ATG9A* and *HSPB8* siRNAs or control siRNA, indicating that *RAB7* and *LC3* were essential for EGFR degradation.

**Table 2:**
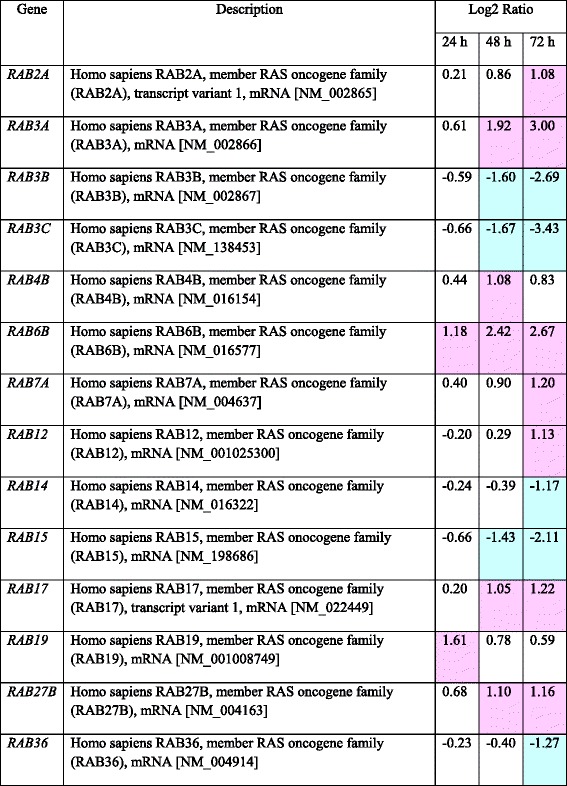
Change of expression levels of *RAB* related genes in MKN28 cells after PFL treatment

Total RNA from PFL-treated and untreated MKN28 cells was extracted and subjected to Aligent Expression Array Analysis with SurePrint G3 Human GE 8x60K v2 (Takara Bio, Japan). The expression level of each gene at each time was determined and compared with that of control RNA from PFL-untreated cells. Highlighting indicates more than a 2-fold change in the expression level.

#### PFL changes the sensitivity of cancer cells to anti-cancer drugs

**Fig. 6:**
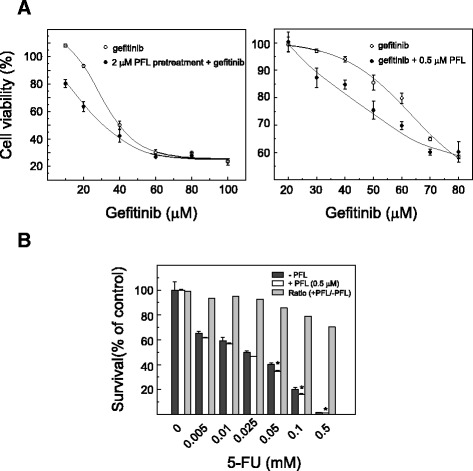
Enhanced sensitivity of cancer cells to anti-cancer drugs following PFL treatment. **a** Enhanced sensitivity of MKN28 cells to gefitinib following PFL treatment. Graphs display the viability of MKN28 cells treated with 2 μM PFL for 24 h, then incubated with various concentration of gefitinib for 72 h. Cell viability was determined by a conventional MTS assay. A representative experiment is shown. Effect of co-incubation of gefitinib with 0.5 μM PFL was evaluated (right panel) in the same manner. **b** Enhanced sensitivity of HT-29 Luc cells to 5-FU by PFL treatment. The cells were incubated with various concentration of 5-FU for 96 h in the presence or absence of 0.5 μM PFL. Histograms represent the cell viability as determined by CellTiter-Glo luminescent cell viability assay. Asterisks indicate values significantly different (*p* < 0.01) between PFL-treated and non-treated cells

The remarkable EGFR downregulation caused by PFL was expected to enhance the effect of EGFR-targeting drugs. To test the adjuvant effect of PFL for gefitinib, a selective inhibitor of EGFR tyrosine kinase, MKN28 cells were pretreated with 2 μM PFL for 24 h prior to gefitinib administration. After removing the medium containing PFL, the cells were incubated with various concentrations of gefitinib for 72 h. As shown in Fig. 6a (left panel), the cancer cells pretreated with PFL showed increased sensitivity to gefitinib at 10 and 20 μM. Co-incubation of 0.5 μM PFL with gefitinib also enhanced sensitivity, although PFL at this concentration was not cytotoxic by itself (Fig. 6a, right panel). To further explore the adjuvant effect of PFL, we tested the 5-FU sensitivity of another cancer cell line, HT-29 Luc cells, in the presence or absence of PFL. As shown in Fig. 6b, 0.5 μM PFL was not harmful to HT-29 Luc by itself, however, 5-FU sensitivity of the cells was increased in the presence of PFL.

#### PFL exhibits an in vivo anti-tumor effect in a mouse model

**Fig. 7:**
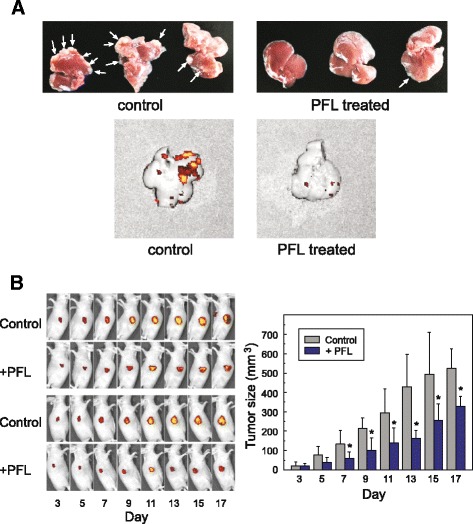
**a** *In vivo* tumor formation assay using PFL treated or non-treated MKN28-EGFP cells. MKN28-EGFP cells were incubated with 10 μM PFL for 3 h and injected to the portal vein of mice. Tumor colonies formed in the liver were detected 4 weeks post-injection. Photographs show the dissected livers (upper panels). Tumor colonies are indicated by white arrows for control (left) and PFL-treated groups (right). Representative IVIS images are shown at the bottom. **b** *In vivo* effect of PFL on tumor growth in nude mice. MKN28 cells were subcutaneously injected to mice and tumors were allowed to grow. PFL (100 μg) in 100 μL PBS was intratumorally-injected three times 1, 3, and 7 days following tumor cells implantation. The histogram displays tumor volume, represented as means ± SD (n = 10). Asterisks indicate values significantly different (*p* < 0.01) between PFL-treated mice and the control. Representative IVIS images are shown for PFL-treated mice and the control

To test the *in vivo* effect of PFL, we employed a mouse model of liver metastasis using portal vein injection of MKN28-EGFP cells. Prior to tumor cell implantation, the cells for PFL treated groups were incubated with 10 μM PFL for 3 h, the same condition used in the *in vitro* adhesion assay described above, then washed with medium to remove unbound lectin. Aliquots of the cells (1 × 10^6^ cells) were injected into the portal vein of BALB/c nude mice and, 4 weeks after implantation, tumor colonies formed in the dissected liver were observed by fluorescence derived from constitutively expressed EGFP in MKN28 cells with IVIS. As shown in Fig. 7a, tumor formation at the liver was inhibited significantly in PFL-treated groups. This effect might be partly attributable to the impaired cell adherence strength of MKN28-EGFP cells to the liver tissue, where collagen IV is abundant. Other factors, such as a dysfunction of cell proliferation, could be involved in PFL-induced tumor suppression in the liver.

Finally, the *in vivo* effect of PFL on the growth of subcutaneous MKN28 tumors in BALB/c nude mice was evaluated. PFL (100 μg) in PBS was injected three times intratumorally 1, 3 and 7 days after tumor implantation. As shown in Fig. 7b, tumor volumes or fluorescence derived from EGFP expressed in MKN28 cells in PFL-treated groups was dramatically decreased, indicating that the growth of tumors, compared with the control, was inhibited by PFL treatment. In addition, no severe symptoms or toxicity, such as decrease in body weight, was observed following PFL treatment.

## Discussion

In the present study, we demonstrated that the high mannose binding bacterial lectin PFL induced rapid intracellular trafficking of both EGFR and α2 integrin in MKN28 gastric cancer cells. The adherence strength of MKN28 cells to ECM was dramatically abolished by PFL treatment, correlating with the loss of cell surface integrins. Activated EGFR transiently modulates α2 integrin cell surface expression and stimulates integrin trafficking via caveolae/raft-mediated endocytosis [9]. Furthermore, physical and functional association between integrins and EGFR also has been demonstrated [10, 11]. Such cross-talk between integrins and EGFR has been hypothesized to play a critical role in cancer progression [12]. It is still unclear whether PFL bound to glycans of integrin and EGFR independently, or to those of integrin/EGFR complexes. In either case, we assume that both molecules internalized by PFL would be eventually degraded, resulting in dysfunction of cell attachment and proliferation. Since high mannose EGFR has reported to represent a novel tumor-specific antigen [13], PFL would be useful as a cancer targeting material. Recently, β1 integrin-silenced cells were reported to exhibit defective activation of EGFR signaling, leading to increased sensitivity to gefitinib [14]. Similarly, we observed that PFL enhanced the susceptibility of MKN28 cells to gefitinib, possibly due to EGFR downregulation. Moreover, enhanced drug sensitivity of other cancer cell lines in the presence of PFL also was observed. For instance, the 5-FU sensitivity of HT29 colon cancer cells was increased in the presence of 0.5 μM PFL, a concentration that was not harmful to the cells, although the effect of PFL was limited compared with EGFR drugs. The mechanism behind this observation is yet to be clarified; however, it is plausible that PFL might activate endocytosis transiently during integrin/EGFR internalization, thereby facilitating uptake of extracellular substances.

Our data suggest that the internalized integrin/EGFR associated with PFL was degraded by autophagy, a mechanism that eliminates damaged proteins, aged proteins and organelles, or harmful aggregated proteins [15–17]. Expression of many autophagic proteins in MKN28 cells was elevated significantly by PFL treatment, but expression of activated apoptotic proteins was hardly detectable. Although we could not rule out the possibility that both autophagy and apoptosis might occur in PFL-treated cancer cells, because both phenomena are reported to cross-talk through shared molecules such as ATG5 by taking either the calpain-cleaved or non-cleaved form [18, 19], the present data indicate that PFL primarily induces autophagy.

Some plant lectins are reported to induce autophagy in cancer cells via a mitochondria-mediated pathway [20]. For example, legume lectin Concanavalin A (ConA), which recognizes high mannose N-glycans as well as monosaccharides including mannose, binds to cell membrane glycoproteins and is internalized and accumulates in mitochondria, resulting in mitochondrial dysfunction and induction of autophagy to degrade these mitochondria [21]. Polygonatum cyrtonema lectin, which belongs to the GNA-related lectin family, induces cancer cell autophagy by promoting the ROS-p38-p53 pathway [22]. By contrast, PFL-induced autophagy is mediated by integrin/EGFR internalization, a mechanism that has not been reported so far, although other surface glycoproteins with high mannose glycans might be involved in this process as well.

Following PFL-induced receptor endocytosis, the association of EGFR with vesicular trafficking proteins such as RAB7 was observed. RAB7, a member of small RAB GTPase family, is known as a common modulator in endocytosis and autophagy. It is involved not only in the maturation of endosomes and autophagosomes, but also in the trafficking of cargos along microtubules and in fusion with lysosomes [15, 23–25]. Furthermore, it is reported that functional RAB7 is required for the degradation of EGFR by the lysosome [26]. In the present study, we observed increased expression of RAB7, and that siRNA against *RAB7* inhibited PFL-induced EGFR degradation, which suggest that RAB7 plays an important role in this degradation process. In addition, ATG9, a multipass transmembrane protein necessary for optimal autophagy [27], increased in expression in response to PFL treatment of MKN28 cells, and colocalized with the internalized EGFR. Mammalian ATG9 has been suggested to interact transiently with isolation membranes and autophagosomes, but its actual function is still controversial [28]. Although the detailed mechanism of receptor membrane trafficking remains be clarified, DNA microarray data suggest that multiple molecules, including autophagic proteins, membrane trafficking proteins, and motor proteins such as dynein, might be involved in PFL-mediated receptor degradation. The roles of autophagy are assumed to differ in different stages of cancer development [29]. For example, autophagy initially has a preventive role against cancer, but for developed cancer cells, it can be utilized to promote cancer cell survival. Although we have no direct evidence as to whether autophagy might contribute to PFL-induced cell death and tumor suppression, it would be plausible that PFL-induced autophagy ultimately affects cell survival, because it leads directly to defects in integrin and EGFR signaling that are crucial for cell adhesion to appropriate location, migration and proliferation.

Direct administration of PFL to subcutaneous tumors significantly reduced tumor growth *in vivo*. Other lectins, such as ESA-2, which is from the same lectin family, reportedly exhibit potent anti-cancer activity when administered intravenously or intraperitoneally in surface-bound form in Span 80 vesicles to mice bearing subcutaneous tumors [30]. Since this lectin family of proteins exhibit a characteristic stable structure, i.e., a domain swap structure with abundant β sheets [3, 7], it would be advantageous to utilize them for clinical applications. Furthermore, it is noteworthy that PFL enhanced the susceptibility of cancer cells to anti-cancer drugs such as gefitinib. Serum levels of surfactant protein D (SP-D), a C-type lectin with innate immune functions, also reportedly are associated with gefitinib efficacy [31]. Therefore, future work will be directed toward evaluating the *in vivo* adjuvant effect of PFL with various anti-cancer drugs to increase therapeutic efficacy and reduce side effects.

## Conclusion

Bacterial lectin PFL exhibited novel anti-cancer characteristics as follows: 1) PFL had a cytotoxic effect on cancer cells, inducing significant autophagy, which was triggered by direct binding of PFL to cell surface integrin/EGFR, causing internalization. PFL would thus be useful as an autophagy stimulator to gain insights into the molecular basis of autophagy in cancer cells. 2) Down-regulation of cell surface integrin by PFL resulted in impaired cell adhesion, and PFL treatment effectively inhibited tumor formation in the liver, suggesting that PFL might inhibit cancer metastasis. 3) Down-regulation of cell surface EGFR by PFL rendered cancer cells susceptible to gefitinib, suggesting that PFL potentially would be useful as an adjuvant with other anti-cancer drugs.

## Additional file

Additional file 1: Table e1. Aligent Expression Array Analysis with SurePrint G3 Human GE 8x60K v2 (Takara Bio, Japan). (XLS 9492 kb)

## Abbreviations

ECM: extracellular matrix
EGFP: enhanced green fluorescent protein
EGFR: epidermal growth factor receptor
FBS: fetal bovine serum
PBS: phosphate buffered saline
PFL: *Pseudomonas fluorescens* lectin
